# Concomitant Transcatheter Edge-to-Edge Repair and Left Atrial Appendage Occlusion

**DOI:** 10.3390/jcm14072257

**Published:** 2025-03-26

**Authors:** Graeme Prosperi-Porta, Adam Dryden, Donna Nicholson, Mark Hynes, Vincent Chan, Richard G. Jung, Pietro Di Santo, Trevor Simard, Marino Labinaz, Benjamin Hibbert, Omar Abdel-Razek

**Affiliations:** 1 Division of Cardiology, University of Ottawa Heart Institute, Ottawa, ON K1Y 4W7, Canada; 2 Division of Cardiac Anesthesiology, University of Ottawa Heart Institute, Ottawa, ON K1Y 4W7, Canada; 3 Division of Cardiac Surgery, University of Ottawa Heart Institute, Ottawa, ON K1Y 4W7, Canada; 4 Department of Cardiovascular Medicine, Mayo Clinic, Rochester, MN 55905, USA

**Keywords:** mitral regurgitation, atrial fibrillation, transcatheter edge-to-edge repair, left atrial appendage occlusion

## Abstract

**Background/Objectives**: Atrial fibrillation is a frequent comorbidity amongst patients undergoing mitral valve transcatheter edge-to-edge repair (M-TEER) for mitral regurgitation. Left atrial appendage occlusion (LAAO) can be performed to reduce the risk of stroke in patients with atrial fibrillation. Both procedures require large-bore venous access, transseptal puncture, and real-time imaging of the left atrium. However, limited data exist evaluating the safety and feasibility of concomitant M-TEER and LAAO. **Methods**: We performed a retrospective review of all concomitant M-TEER and LAAO procedures at our institution between May 2019 and September 2024 to evaluate the safety and feasibility of this approach. **Results**: Concomitant left atrial appendage occlusion was successful in all 15 patients, requiring an additional 15 min (IQR 11–29) of procedural time. No patients died or had a major vascular complication. Routine transesophageal echocardiography performed within 90 days showed no device related thrombus, and no significant peri-device leak in any patients. **Conclusions**: Concomitant M-TEER and LAAO are feasible but additional prospective studies or randomized trials are needed to evaluate the potential clinical benefit.

## 1. Introduction

Mitral valve transcatheter edge-to-edge repair (M-TEER) is commonly used to treat patients with severe symptomatic mitral regurgitation (MR). Randomized studies have demonstrated M-TEER’s safety and efficacy, with current guidelines endorsing its use in patients with severe symptomatic functional and degenerative MR at high surgical risk despite maximally tolerated guideline-directed medical therapy [1,2,3]. Between 2014 and 2019, M-TEER volume in the United States has increased nearly 10-fold from 1152 per year to 10,460 per year, with this number continuing to grow [4].

Atrial fibrillation (AF) is a frequent comorbidity amongst patients with MR and is present in 48–63% of patients undergoing M-TEER [1,3,5]. Patients with AF have an increased morbidity and mortality that is irrespective of symptomatology [6]. Despite similar procedural success, patients with AF undergoing M-TEER have higher 1-year mortality, heart failure hospitalizations, stroke, and bleeding compared to patients with a sinus rhythm [5]. Additionally, patients undergoing M-TEER frequently have contraindications to oral anticoagulant (OAC) or have an excessively high estimated bleeding risk. Left atrial appendage occlusion (LAAO) represents an alternative to chronic OAC use that is associated with decreased all-cause mortality and a reduction in the composite of death, hemorrhagic, and thromboembolic events compared to direct OACs [7].

M-TEER and LAAO share technical similarities with both procedures requiring large-bore femoral venous access, transseptal puncture, and real-time imaging of the left atrial structures. During atrial fibrillation ablation where the left atrium is accessed via a transseptal puncture, concomitant LAAO was shown to be technically successful in 98.8% of patients with a low 0.6% rate of serious adverse events [8]. Therefore, concomitant LAAO with M-TEER is an attractive option for patients with indications for both procedures to simplify recovery time, reduce cost and mitigate the cumulative risks associated with vascular access and transseptal puncture when performed on separate occasions. However, limited data exist definitively evaluating the feasibility and safety of this approach. The aim of this study is to report our experience with concomitant M-TEER and LAAO.

## 2. Materials and Methods

All patients that underwent M-TEER at the University of Ottawa Heart Institute between May 2019 and September 2024 were retrospectively reviewed for inclusion. Baseline clinical characteristics, procedural characteristics, and clinical outcomes were obtained from electronic medical records. The decision to perform concomitant M-TEER and LAAO was made after a discussion between the treating clinician and the patient prior to the procedure. All cases were reviewed by a heart team for clinical and anatomic suitability for M-TEER and LAAO if applicable.

### 2.1. Procedural Characteristics

Patients underwent M-TEER under general anesthesia with fluoroscopic and transesophageal echocardiogram (TEE) guidance. All procedures are performed via the right femoral vein, with a transseptal puncture performed using a radiofrequency needle (Baylis Medical, Mississauga, ON, Canada). M-TEER was performed using the MitraClip™ system (Abbott Vascular, Santa Clara, CA, USA). LAAO procedures were performed using the Amulet™ device (Abbott Vascular, Santa Clara, CA, USA), the Amplatzer™ Cardiac Plug device (Abbott Vascular, Santa Clara, CA, USA), or the WATCHMAN FLX™ device (Boston Scientific, Marlborough, MA, USA).

**Figure 1:**
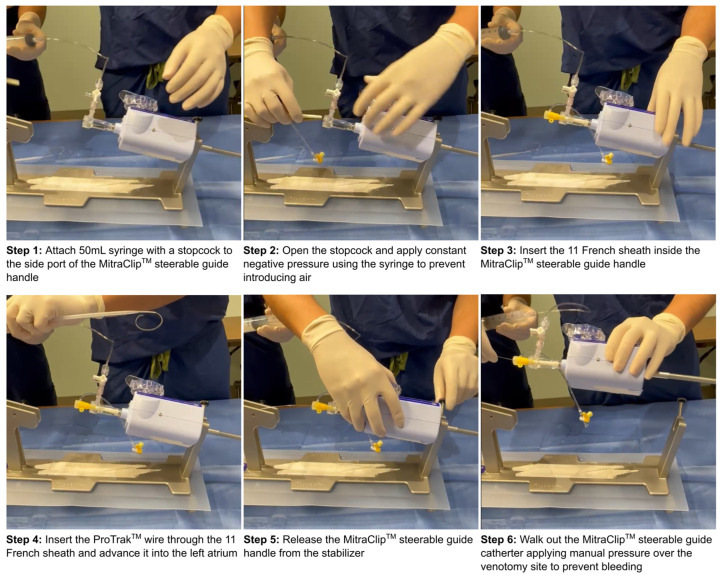
Method used for exchanging the MitraClip™ steerable guide with an 45° × 45° TorqVue™ delivery sheath.

After successful MitraClip™ delivery, LAAO was performed as follows (Figure 1). An 11 French sheath was placed in the lumen of the MitraClip™ device steerable guide catheter. A ProTrack™ pigtail wire (Boston Scientific, Marlborough, MA, USA) was then advanced into the left atrium and the steerable guide catheter was removed over the wire. An 18 Fr sheath (Cook Medical, Bloomington, IN, USA) was then placed in the femoral vein for hemostasis. Next, a 45° × 45° TorqVue™ delivery sheath (Abbott Vascular, Santa Clara, CA, USA) was advanced over the ProTrack™ wire through the original M-TEER transseptal puncture. The LAAO device was then sized using TEE and deployed under TEE and fluoroscopic guidance. Following implantation, vascular access was closed using either an Abbot Perclose Proglide™ (Abbott Vascular, Santa Clara, CA, USA) or a figure of eight suture at the operator’s discretion. Transthoracic echocardiograms were performed prior to discharge, with routine TEEs performed within 90 days to assess for peri-device leak (PDL) and device related thrombus (DRT).

### 2.2. Outcomes

The following procedural outcomes were assessed: procedural success, total procedural time, contrast volume used, time from steerable guide sheath removal to LAAO device implantation, bleeding as assessed by the Bleeding Academic Research Consortium classification system, and device related complications. Major adverse cardiovascular events including all-cause death, cardiovascular death, stroke, transient ischemic attack, systemic embolization, myocardial infarction, and bleeding at the 1-year mark were assessed.

### 2.3. Statistical Analysis

Mean ± standard deviation (SD) or median with interquartile range (IQR) are reported for continuous variables according to normality, and absolute and relative frequencies are reported for categorical variables.

## 3. Results

A total of 356 M-TEER cases were performed between May 2019 and September 2024. Of these, 15 patients underwent concomitant LAAO during the index M-TEER intervention (Table 1). The median age was 80 years (IQR 76–85) with three females (20.0%). There were eight patients (53%) with a degenerative mechanism for their MR. The median CHA_2_DS_2_-VASc and HAS-BLED scores were 5 (IQR 4–6) and 3 (IQR 3–3), respectively.

**Figure 2:**
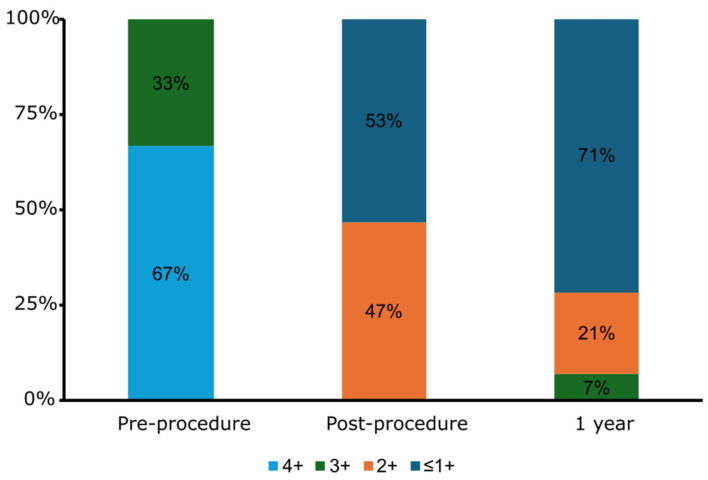
Mitral regurgitation severity before MitraClip™, immediately post-procedure, and at 1 year follow-up.

Procedural success was achieved in 100% of cases (Table 2). All M-TEERs were performed using the MitraClip™ system and in 73% of cases LAAO was performed using the Amulet™ device. The median time from removal of the MitraClip™ guide to LAAO deployment was 15 min (IQR 11–29). Mitral regurgitation, which was moderate–severe in 5 patients (33%) and severe in 10 patients (67%), was reduced to ≤moderate in severity in all patients (Figure 2). Most patients received dual antiplatelet therapy (60%) following the procedure and the remainder received a direct oral anticoagulant (DOAC) (Table 3). No patients died or had vascular complications during the index hospitalization. One patient had a post-procedure hemorrhagic pericardial effusion requiring urgent pericardiocentesis without further intervention needed. There were no leaflet detachments or device embolizations. At the routine follow-up TEE there was no DRT detected, and no patients had a significant PDL.

At the one-year follow-up, there was one patient (7%) who died from ventricular tachycardia, one (7%) major bleeding event due to radiation cystitis, and six (40%) patients required rehospitalization, with only two (13%) attributable to cardiovascular causes (both due to ventricular tachycardia) (Table 4). There was no stroke, transient ischemic attack or systemic embolism, and no myocardial infarction. No patients required repeat mitral valve intervention, LAAO intervention, or unplanned cardiac surgery. At the 1-year follow-up, the majority of patients (71%) had mild (1+) or less MR and only one patient had moderate–severe (3+) MR (7%) on transthoracic echocardiography (Figure 2). At the 1-year follow-up, 70% of patients remained off of full dose anticoagulation, with the majority taking aspirin monotherapy (36%) or no antithrombotic therapy (29%) (Table 3).

## 4. Discussion

Our single-center case series supports the growing practice of concomitant LAAO after M-TEER. We identified several important findings. First, 100% of patients had a successful LAAO implant after M-TEER, with no significant PDL at follow-up. Although transseptal punctures performed for M-TEER may not be perfectly suited to LAAO, this study along with others suggests the feasibility of this approach with high implant success rates [9,10]. Second, concomitant LAAO after M-TEER is safe with only one patient developing a post procedural pericardial effusion. Third, performing LAAO after M-TEER adds only 15 min of additional procedure time, making it logistically feasible in most practices.

LAAO has an expanding body of evidence supporting the safety and efficacy of this procedure, with a rapid growth of implants in the United States [11]. With AF being present in up to 63% of patients undergoing M-TEER for both functional and degenerative MR [12], concomitant LAAO is an attractive option in selected patients [5]. Current evidence would suggest that concomitant LAAO is performed mostly with catheter pulmonary vein isolation or transcatheter aortic valve replacement (TAVR) but less commonly during M-TEER [9]. The WATCH-TAVR randomized trial found that despite the additional exposure to large-bore venous access, transseptal puncture, and increased general anesthetic use concomitant TAVR with LAAO was non-inferior to TAVR with medical therapy, with low rates of procedural complications [13]. However, the procedural similarities between LAAO and M-TEER, including large-bore femoral venous access, transseptal puncture, and general anesthesia, make concomitant LAAO and M-TEER an attractive option for select patients. A single concomitant procedure reduces anesthetic exposure, total recovery time, and is associated with a shorter hospital total length of stay and lower costs compared to sequential procedures with similar adverse events and in-hospital mortality [9]. Based on Canadian registry data, isolated LAAO requires an average of 89 min of procedural time, which in our study was reduced to only 15 min when performed immediately following M-TEER [14].

There are limited prospective or randomized data evaluating concomitant transcatheter procedures with LAAO. Even fewer studies have reported the safety and feasibility of concomitant M-TEER with LAAO, with the majority of data limited to small case series and case reports [10,15,16,17,18,19]. The WATCH-TEER study is the only prospective evaluation of concomitant LAAO and M-TEER, with only 24 patients included [19]. Similarly to our study, LAAO implant success was 100% and required approximately 20 min of additional procedural time without any major procedure related complications, and only two patients had a small PDL and one patient had DRT in follow-up. In another retrospective multi-center cohort study that included 30 patients, LAAO implant success was 93% with two peri-procedural complications (1 death from cardiac tamponade and 1 peri-procedural transient ischemic attack), and only 1 patient had a PDL and none had DRT in follow up [10]. The lower LAAO implant success in this study may be due to earlier experiences with the concomitant LAAO and M-TEER or lower procedural volumes (average of ~3 performed at each site between 2015 and 2019). We identified no significant PDL > 3 mm or DRT in our cohort at the routine follow-up TEE performed within 90 days. Importantly, small PDL < 3 mm have been shown to regress with time, suggesting long-term durability of these results [20]. However, the timing of DRT is heterogeneous with 42% occurring < 90 days, 57% occurring between 90 and 365 days, and 1% occurring after 365 days [21]. While it is possible that some patients developed subclinical DRT after undergoing the post-procedure TEE, we importantly saw no embolic events during the 1-year follow-up. These findings suggest that using a common transseptal puncture location does not negatively impact the positioning of the LAAO device, although larger studies are needed.

The development of a pericardial effusion is a serious complication from LAAO. The incidence of a significant pericardial effusion requiring intervention after LAAO is reported to be approximately 1.3%, with risk factors including advanced age, higher CHA_2_DS_2_-VASC score, obesity, female sex, left ventricular dysfunction, paroxysmal atrial fibrillation, prior bleeding, lower serum albumin, pre-procedural dual antiplatelet therapy, sinus rhythm during the procedure, and moderate sedation rather than general anesthesia [22,23]. We had one patient that developed a hemorrhagic pericardial effusion requiring pericardiocentesis. Their only risk factor was a history of prior recurrent epistaxis and the LAAO implanted was appropriately sized for this patient’s left atrial appendage orifice based on real-time TEE. Studies with larger sample sizes or pooled multi-center data are needed to determine whether the risk of pericardial effusion is higher with concomitant M-TEER with LAAO.

Chronic AF is associated with worse clinical outcomes among patients undergoing other cardiac procedures including coronary revascularization and transcatheter aortic valve replacement [24,25]. Similarly, patients with AF undergoing M-TEER have worse clinical outcomes despite similar procedural success [5,26]. One potential explanation may be due to increased bleeding in AF from anticoagulation use. Due to the lack of large registries or randomized trials, is not yet known whether concomitant LAAO during M-TEER could reduce bleeding events in patients with AF. We observed major bleeding at 1-year in only one patient (7%), which is in keeping with the 3.3% to 25% reported in other studies [10,19]. In patients without contraindications to anticoagulation or excessive bleeding risk it is unknown whether clinical risk prediction tools such as the CHA_2_DS_2_-VASc score [27] or more advanced techniques such as speckle-tracking echocardiography of the left atrium and appendage [28] could identify patients at higher stroke risk who might benefit from LAAO. The ongoing Fourth Left Atrial Appendage Occlusion Study (NCT05963698) may provide some insight. The presence of both MR and AF is undoubtedly worse than either in isolation and without comprehensive prognostic studies this population’s numerous confounding comorbidities makes comparing outcomes challenging [29].

In the absence of randomized control trials, observational data that confers safety and technical success are currently the only guiding information when considering concomitant M-TEER and LAAO in patients. Future large prospective cohorts, pooled multi-center data, or randomized trials are needed to confirm the suspected benefit of concomitant LAAO and M-TEER.

Our study has several limitations. First, the sample size is small and includes only a single center. At our site, two experienced structural interventional cardiologists performed over 350 M-TEER and 250 total LAAO implants during the study period between 2019 and 2024. The same results may not be achieved at lower volume centers or by less experienced operators. Definitive conclusions on the safety and efficacy of concomitant procedures will require larger multi-center cohorts or randomized studies. Second, as a retrospective observational cohort study there may be unmeasurable selection bias related to the patients who underwent concomitant M-TEER and LAAO. All patients were reviewed by a multidisciplinary team of structural interventional cardiologists, cardiac surgeons, anesthesiologists, and echocardiographers before acceptance and only patients with anatomic suitability for both interventions from a common transseptal puncture were accepted for the concomitant intervention. Therefore, concomitant M-TEER and LAAO should only be considered in select patients with indications for both procedures after a detailed multidisciplinary anatomic review. Before widespread adoption can take place, additional prospective or randomized data evaluating the feasibility and safety of concomitant M-TEER and LAAO in a broader population are needed. Unfortunately, no studies are currently underway. Finally, we used only the MitraClip™ system for M-TEER and most patients at our center received the Amulet™. As such, the findings cannot be generalized to other technologies available for M-TEER and LAAO.

## 5. Conclusions

Atrial fibrillation is a common comorbidity in patients with severe symptomatic MR undergoing M-TEER. Concomitant LAAO can be performed safely and effectively at the time of M-TEER. Larger prospective cohort studies, pooled multi-center data, or randomized studies are needed to more definitively evaluate the feasibility and safety of concomitant M-TEER and LAAO and to determine which patients may benefit most.

## Figures and Tables

**Table 1:** Baseline patient characteristics.

	M-TEER + LAAO (*n* = 15)
Age, Years (IQR)	80.0 (76.0–85.0)
Female, *n* (%)	3 (20%)
Prior Myocardial Infarction, *n* (%)	4 (27%)
Diabetes, *n* (%)	5 (33%)
Hypertension, *n* (%)	10 (67%)
Dyslipidemia, *n* (%)	6 (40%)
Smoking, *n* (%)	3 (20%)
Creatinine (mg/dL)	1.4 (1.1–2.5)
Prior Stroke/CVA, *n* (%)	3 (20%)
Peripheral Arterial Disease, *n* (%)	1 (7%)
Chronic Obstructive Pulmonary Disease, *n* (%)	2 (13%)
Left Ventricular Ejection Fraction, % (IQR)	45% (40–55%)
Mitral Regurgitation Mechanism	
Degenerative, *n* (%)	8 (53%)
Functional, *n* (%)	7 (47%)
Mitral Regurgitation Severity	
3+	5 (33%)
4+	10 (67%)
Atrial Fibrillation	
Permanent, *n* (%)	10 (67%)
Paroxysmal, *n* (%)	5 (33%)
NYHA Class	
II, *n* (%)	9 (6%)
III, *n* (%)	6 (40%)
IV, *n* (%)	0 (0%)
CHA_2_DS_2_-VaSC Score	5 (4–6)
HAS-BLED Score	3 (3–3)

**Table 2:** Procedural characteristics.

	M-TEER + LAAO (*n* = 15)
Total procedure time, min (IQR)	111 (93–124)
Total fluoroscopy time, min (IQR)	31 (22–45)
General anesthesia	15 (100%)
Transesophageal guidance	15 (100%)
M-TEER Procedure	
Procedure completed	15 (100%)
Duration of procedure, min (IQR)	85 (80–104)
#of MitraClip™ deployed per case	
1	9 (60)
2	3 (20)
3	3 (20)
Type of devices deployed	
XT	0 (0%)
NT	5 (21%)
XTW	7 (29%)
NTW	12 (50%)
Residual MR	
≤1+	9 (53%)
2+	6 (47%)
3+	0 (0%)
4+	0 (0%)
Post-M-TEER mitral valve gradient, mmHg (IQR)	3.9 (3.4–4.5)
LAAO Procedure	
MitraClip™ sheath removal to LAAO deployment, min (IQR)	15 (11–29)
LAAO device used	
Amplatzer™ Cardiac Plug	2 (13.3)
WATCHMAN™ FLX	2 (13.3)
Amulet™	11 (73.3)
Device margin leak > 3 mm	0 (0%)
ASD Closure	4 (27%)

ASD = atrial septal defect; MR = mitral regurgitation; M-TEER = mitral valve transcatheter edge-to-edge repair; LAAO = left atrial appendage occlusion.

**Table 3:** Baseline and follow-up antithrombotic therapy.

	Baseline (*n* = 15)	Post-Procedure (*n* = 15)	1 Year (*n* = 14)
Aspirin	2 (13%)	0	5 (36%)
P2Y12	0	0	0
DAPT	2 (13%)	9 (60%)	1 (7%)
Warfarin	0	0	0
LMWH	0	0	1 (7%)
DOAC	8 (53%)	6 (40%)	3 (21%)
No antithrombotic therapy	3 (20%)	0	4 (29%)

DAPT = dual antiplatelet therapy; LMWH = low molecular weight heparin; DOAC = direct oral anticoagulant.

**Table 4:** Clinical outcomes at 45 days and 1-year follow-up.

	0–45 Days ^a^ (*n* = 15)	45 Days–1 Year (*n* = 15)	Cumulative Insidence at 1 Year (*n* = 15)
All-cause death	0	1 (7%)	1 (7%)
Cardiac death	0	1 (7%)	1 (7%)
Non-cardiac death	0	0	0
HF hospitalization	0	0	0
Major bleeding	0	1 (7%)	1 (7%)
Life threatening bleeding	1 (7%)	0	1 (7%)
Stroke or TIA	0	0	0
Hemorrhagic stroke	0	0	0
Vascular complications	0	0	0
Myocardial infarction	0	0	0
New dialysis	0	0	0
Endocarditis	0	0	0
Mitral valve-related intervention	0	0	0
Unplanned cardiac intervention or surgery	0	0	0
M-TEER embolization	0	0	0
LAAO-related intervention	0	0	0
LAAO thrombus	0	0	0
LAAO migration	0	0	0
LAAO embolization	0	0	0

Values are *n* (%). ^a^ Including procedural outcomes. HF = heart failure; LAAO = left atrial appendage occlusion; M-TEER = mitral valve transcatheter edge-to-edge repair; TIA = transient ischemic attack.

## Data Availability

The original contributions presented in this study are included in the article. Further inquiries can be directed to the corresponding author.

## Abbreviations

The following abbreviations are used in this manuscript:

AF	Atrial fibrillation
DRT	Device related thrombus
LAAO	Left atrial appendage occlusion
M-TEER	Mitral transcatheter edge-to-edge repair
MR	Mitral regurgitation
OAC	Oral anticoagulant
PDL	Peri-device leak
TEE	Transesophageal echocardiogram

## References

[1] Stone G.W., Abraham W.T., Lindenfeld J., Kar S., Grayburn P.A., Lim D.S., Mishell J.M., Whisenant B., Rinaldi M., Kapadia S.R. (2023). Five-Year Follow-up after Transcatheter Repair of Secondary Mitral Regurgitation. N. Engl. J. Med..

[2] Otto C.M., Nishimura R.A., Bonow R.O., Carabello B.A., Erwin J.P., Gentile F., Jneid H., Krieger E.V., Mack M., McLeod C. (2021). 2020 ACC/AHA Guideline for the Management of Patients With Valvular Heart Disease: A Report of the American College of Cardiology/American Heart Association Joint Committee on Clinical Practice Guidelines. Circulation.

[3] Anker S.D., Friede T., Bardeleben R.-S.v., Butler J., Khan M.-S., Diek M., Heinrich J., Geyer M., Placzek M., Ferrari R. (2024). Transcatheter Valve Repair in Heart Failure with Moderate to Severe Mitral Regurgitation. N. Engl. J. Med..

[4] Mack M., Carroll J.D., Thourani V., Vemulapalli S., Squiers J., Manandhar P., Deeb G.M., Batchelor W., Herrmann H.C., Cohen D.J. (2021). Transcatheter Mitral Valve Therapy in the United States: A Report From the STS-ACC TVT Registry. J. Am. Coll. Cardiol..

[5] Arora S., Vemulapalli S., Stebbins A., Ramm C.J., Kosinski A.S., Sorajja P., Piccini J.P., Cavender M.A., Vavalle J.P. (2019). The Prevalence and Impact of Atrial Fibrillation on 1-Year Outcomes in Patients Undergoing Transcatheter Mitral Valve Repair: Results From the Society of Thoracic Surgeons/American College of Cardiology Transcatheter Valve Therapy Registry. JACC Cardiovasc. Interv..

[6] Karakasis P., Pamporis K., Siontis K.C., Theofilis P., Samaras A., Patoulias D., Stachteas P., Karagiannidis E., Stavropoulos G., Tzikas A. (2024). Major clinical outcomes in symptomatic vs. asymptomatic atrial fibrillation: A meta-analysis. Eur. Heart J..

[7] Fernandes J.M., Pinheiro R.P.S., Serpa F., de Andrade N.M., Pereira V., Sbardelotto Â.E.E., Gomes W.F. (2025). Left atrial appendage occlusion devices vs direct oral anticoagulants for atrial fibrillation: An updated systematic review and meta-analysis. Curr. Probl. Cardiol..

[8] Wazni O.M., Saliba W.I., Nair D.G., Marijon E., Schmidt B., Hounshell T., Ebelt H., Skurk C., Oza S., Patel C. (2024). Left Atrial Appendage Closure after Ablation for Atrial Fibrillation. N. Engl. J. Med..

[9] Ismayl M., Ahmed H., Freeman J.V., Alkhouli M., Lakkireddy D., Goldsweig A.M. (2024). Safety and Efficacy of Combining Left Atrial Appendage Occlusion With Another Cardiac Procedure. JACC Cardiovasc. Interv..

[10] D’Amico G., Estèvez-Loureiro R., Rofastes X.F., Ronco F., Nombela-Franco L., Melica B., Bedogni F., Saia F., Cruz-Gonzalez I., Tarantini G. (2021). Combined Procedure of Percutaneous Mitral Valve Repair and Left Atrial Appendage Occlusion: A Multicenter Study. JACC Cardiovasc. Interv..

[11] Freeman James V., Varosy P., Price Matthew J., Slotwiner D., Kusumoto Fred M., Rammohan C., Kavinsky Clifford J., Turi Zoltan G., Akar J., Koutras C. (2020). The NCDR Left Atrial Appendage Occlusion Registry. J. Am. Coll. Cardiol..

[12] von Bardeleben R.S., Mahoney P., Morse M.A., Price M.J., Denti P., Maisano F., Rogers J.H., Rinaldi M., De Marco F., Rollefson W. (2023). 1-Year Outcomes With Fourth-Generation Mitral Valve Transcatheter Edge-to-Edge Repair From the EXPAND G4 Study. JACC Cardiovasc. Interv..

[13] Kapadia S.R., Krishnaswamy A., Whisenant B., Potluri S., Iyer V., Aragon J., Gideon P., Strote J., Leonardi R., Agarwal H. (2024). Concomitant Left Atrial Appendage Occlusion and Transcatheter Aortic Valve Replacement Among Patients With Atrial Fibrillation. Circulation.

[14] Saw J., Inohara T., Gilhofer T., Uchida N., Pearce C., Dehghani P., Kass M., Ibrahim R., Morillo C., Wardell S. (2023). The Canadian WATCHMAN Registry for Percutaneous Left Atrial Appendage Closure. CJC Open.

[15] Kuwata S., Taramasso M., Zuber M., Suetsch G., Attinger-Toller A., Wicki D., Maisano F., Nietlispach F. (2017). Feasibility of concomitant MitraClip and left atrial appendage occlusion. EuroIntervention.

[16] Tichelbäcker T., Puls M., Jacobshagen C., Hasenfuß G., Schillinger W., Hünlich M., Schroeter M.R. (2016). MitraClip^®^ and Amplatzer^®^ cardiac plug implantation in a single procedure: A reasonable approach?. Int. J. Cardiol..

[17] Francisco A.R.G., Infante de Oliveira E., Nobre Menezes M., Carrilho Ferreira P., Canas da Silva P., Nobre Â., Pinto F.J. (2017). Combined MitraClip implantation and left atrial appendage occlusion using the Watchman device: A case series from a referral center. Rev. Port. Cardiol..

[18] Freixa X., Estévez-Loureiro R., Carrasco-Chinchilla F., Arzamendi D., Jiménez-Quevedo P., Nombela-Franco L., Cruz-González I., Amat-Santos I.J., Sabaté M. (2017). Initial Results of Combined MitraClipÂ^®^ Implantation and Left Atrial Appendage Occlusion. J. Heart Valve Dis..

[19] Al-Abcha A., Di Santo P., Rihal C.S., Simard T., Hibbert B., Alkhouli M. (2025). Outcomes of Combined Left Atrial Appendage Occlusion and Transcatheter Mitral Edge-to-Edge Repair: The WATCH-TEER Study. JACC Adv..

[20] Afzal Muhammad R., Gabriels James K., Jackson Gregory G., Chen L., Buck B., Campbell S., Sabin Dawn F., Goldner B., Ismail H., Liu Christopher F. (2022). Temporal Changes and Clinical Implications of Delayed Peridevice Leak Following Left Atrial Appendage Closure. JACC Clin. Electrophysiol..

[21] Alkhouli M., Busu T., Shah K., Osman M., Alqahtani F., Raybuck B. (2018). Incidence and Clinical Impact of Device-Related Thrombus Following Percutaneous Left Atrial Appendage Occlusion. JACC Clin. Electrophysiol..

[22] Price M.J., Valderrábano M., Zimmerman S., Friedman D.J., Kar S., Curtis J.P., Masoudi F.A., Freeman J.V. (2022). Periprocedural Pericardial Effusion Complicating Transcatheter Left Atrial Appendage Occlusion: A Report From the NCDR LAAO Registry. Circ. Cardiovasc. Interv..

[23] Munir M.B., Khan M.Z., Darden D., Pasupula D.K., Balla S., Han F.T., Reeves R., Hsu J.C. (2021). Pericardial effusion requiring intervention in patients undergoing percutaneous left atrial appendage occlusion: Prevalence, predictors, and associated in-hospital adverse events from 17,700 procedures in the United States. Heart Rhythm..

[24] Jung R.G., Abdel-Razek O., Di Santo P., Gillmore T., Stotts C., Makwana D., Soriano J., Moreland R., Verreault-Julien L., Goh C.Y. (2022). Impact of atrial fibrillation on the risk of major adverse cardiac events following coronary revascularisation. Open Heart.

[25] Urena M., Webb J.G., Eltchaninoff H., Munoz-Garcia A.J., Bouleti C., Tamburino C., Nombela-Franco L., Nietlispach F., Moris C., Ruel M. (2015). Late cardiac death in patients undergoing transcatheter aortic valve replacement: Incidence and predictors of advanced heart failure and sudden cardiac death. J. Am. Coll. Cardiol..

[26] Feldman T., Foster E., Glower D.D., Kar S., Rinaldi M.J., Fail P.S., Smalling R.W., Siegel R., Rose G.A., Engeron E. (2011). Percutaneous repair or surgery for mitral regurgitation. N. Engl. J. Med..

[27] Friberg L., Rosenqvist M., Lip G.Y. (2012). Evaluation of risk stratification schemes for ischaemic stroke and bleeding in 182 678 patients with atrial fibrillation: The Swedish Atrial Fibrillation cohort study. Eur. Heart J..

[28] Cameli M., Lunghetti S., Mandoli G.E., Righini F.M., Lisi M., Curci V., Tommaso C.D., Solari M., Nistor D., Gismondi A. (2017). Left Atrial Strain Predicts Pro-Thrombotic State in Patients with Non-Valvular Atrial Fibrillation. J. Atr. Fibrillation.

[29] Jung R.G., Stotts C., Gupta A., Prosperi-Porta G., Dhaliwal S., Motazedian P., Abdel-Razek O., Santo P.D., Parlow S., Belley-Cote E. (2024). Prognostic Factors Associated with Mortality in Cardiogenic Shock—A Systematic Review and Meta-Analysis. NEJM Evid..

